# Potential of gut microbiota for lipopolysaccharide biosynthesis in European women with type 2 diabetes based on metagenome

**DOI:** 10.3389/fcell.2022.1027413

**Published:** 2022-10-11

**Authors:** Ying Dong, Pan Wang, Xinchuan Yang, Mulei Chen, Jing Li

**Affiliations:** Heart Center and Beijing Key Laboratory of Hypertension, Beijing Chaoyang Hospital, Capital Medical University, Beijing, China

**Keywords:** enzyme, gut microbiota, lipopolysaccharide, metagenome, type 2 diabetes

## Abstract

The abnormal accumulation of lipopolysaccharide (LPS) plays a crucial role in promoting type 2 diabetes (T2D). However, the capability of the gut microbiota to produce LPS in patients with T2D is still unclear, and evidence characterizing the patterns of gut microbiota with LPS productivity remains rare. This study aimed to uncover the profiles of LPS-biosynthesis-related enzymes and pathways, and explore the potential of LPS-producing gut microbiota in T2D. The gut metagenomic sequencing data from a European female cohort with normal glucose tolerance or untreated T2D were analyzed in this study. The sequence search revealed that the relative abundance of the critical enzymes responsible for LPS biosynthesis was significantly high in patients with T2D, especially for N-acetylglucosamine deacetylase, 3-deoxy-D-manno-octulosonic-acid transferase, and lauroyl-Kdo2-lipid IVA myristoyltransferase. The functional analysis indicated that a majority of pathways involved in LPS biosynthesis were augmented in patients with T2D. A total of 1,173 species from 335 genera containing the gene sequences of LPS enzymes, including LpxA/B/C/D/H/K/L/M and/or WaaA, coexisted in controls and patients with T2D. Critical taxonomies with discriminative fecal abundance between groups were revealed, which exhibited different associations with enzymes. Moreover, the identified gut microbial markers had correlations with LPS enzymes and were subsequently associated with microbial pathways. The present findings delineated the potential capability of gut microbiota toward LPS biosynthesis in European women and highlighted a gut microbiota−based mechanistic link between the disturbance in LPS biosynthesis and T2D. The restoration of LPS levels through gut microbiota manipulation might offer potential approaches for preventing and treating T2D.

## Introduction

Diabetes mellitus (DM) is a well-known risk factor for cardiovascular and cerebrovascular diseases, such as heart failure, myocardial infarction, angina pectoris, stroke, and atrial fibrillation etc., which not only impose huge societal medical burdens but also seriously affect the life quality of suffered patients (Suzuki et al., 2022), especially among women (Jarrett et al., 1982; Barrett-Connor and Ferrara, 1998). Furthermore, previous studies indicated that the highest incidence of newly diagnosed type 2 diabetes (T2D) is between the ages of 45–64 years and 65–79 years, with an estimated 12 new per 1,000 people 45–64 years old, and 15 new patients per 1,000 people at 65–79 years old, respectively. The T2D prevalence is highest among patients older than 65 years, with 27% in 45-year-old compared with 18% in 64-year-old (Lodhi, 2021). The prevalence rate of type 2 diabetes (T2D) in European countries was noted to be in the range of 8%–9% (Altobelli et al., 2020). It was reported that the number of patients with diabetes increased by 3.09% per year in men and 1.92% in women (Altobelli et al., 2020). In China, the prevalence of diabetes increased from 0.7% to 12.8% during 1980–2017. Nowadays, more than a third of adults have been diagnosed with pre-diabetes (Wei et al., 2021).

Gut microbiota is considered to be one of the most crucial factors in the pathology and physiology performance of the host. Over the past few years, the potential role of the gut microbiota in the pathogenesis of T2D has been focused. Substantial evidence has identified gut dysbiotic microbiota in patients with T2D and insulin resistance, even preceding T2D development (Zhong et al., 2019; Wu et al., 2020; Chen et al., 2021; Que et al., 2021; Vals-Delgado et al., 2022). In the individuals diagnosed with T2D after a median of 60 months of follow-up, the preponderance of *Prevotella, Granulicatella, Streptococcus, Sutterella, Actinomyces*, and *Paraprevotella* was detected at the baseline (Vals-Delgado et al., 2022). Various bacterial genera with significant enrichment or depletion in patients with T2D were also identified, such as *Barnesiella, Butyrivibrio, Coprobacter, Tyzzerella, Paraprevotella, Desulfovibrio, Enterobacter*, and *Neisseria* (Que et al., 2021). Moreover, the intestinal taxonomic features at the species level were found to be predictive for incident T2D over the long-term follow-up in Finnish adults (Ruuskanen et al., 2022). Most recently, a population-based longitudinal cohort study in Chinese adults further confirmed the association of gut microbiota with glycemic control and incident T2D, and highlighted the potential of multiple gut microbiota in the diagnosis or therapy for T2D (Wang et al., 2022).

The metabolites of gut microbiota, such as lipopolysaccharide (LPS), trimethylamine-N-oxide (TMAO), and short-chain fatty acids (SCFA), which are the key mediators of microbe–host crosstalk, have been regarded as the main molecules related to T2D (Zhang L et al., 2021). The differences in the abundance of specific gut microbial enzymes for the biosynthesis of TMAO and SCFA have been demonstrated in T2D and other diseases such as atherosclerotic cardiovascular disease (Jie et al., 2017). The pro-inflammatory bacterial product LPS, which significantly increased in women with gestational DM compared with those in late pregnancy (Zhang H et al., 2021), was indicated to contribute to insulin resistance (Cani et al., 2007). The interventions of gut microbiota with kombucha were suggested to reduce the displacement of LPS and inhibit the occurrence of inflammation and insulin resistance for improving hyperglycemia in mice with T2D (Xu et al., 2022). In addition, probiotic *Lactiplantibacillus plantarum* Y15 was documented to alleviate T2D *via* reshaping the structure of gut microbiota and decreasing the abundance of LPS-producing bacteria, subsequently reducing the levels of LPS and pro-inflammatory cytokines (Liu et al., 2022). The clinical strategies to remodel the gut microbiota, and hence to modify the metabolites of microbiota such as LPS, might represent novel targets and future directions for T2D management.

However, the underlying mechanisms of how gut microbiota influences LPS biosynthesis during the progression of T2D are relatively unknown. Thus, the key microbes harboring the critical LPS-biosynthesis-related enzymatic genes and involved in LPS biosynthesis functions were investigated in a cohort of European women with normal glucose tolerance (NGT) or untreated T2D.

## Materials and methods

### Study populations of metagenomic analysis

All 70-year-old women identified through the Swedish Gothenburg County registry (Brohall et al., 2006; Karlsson et al., 2013), were invited to participate in the previous research (Karlsson et al., 2013). The public metagenomic sequence datasets of the present study cohort were obtained from previously reported research with European women (Karlsson et al., 2013). The accession code was Sequence Read Archive ERP002469. The individuals with NGT or T2D were included in the present study. A restriction criterion was applied to investigate the information on gut microbiota producing LPS in patients with T2D and healthy women. Participants were invited to take screening examinations including oral glucose tolerance tests (OGTT). Women with known diabetes who were taking oral antidiabetic drugs or insulin did not have to undergo OGTT, whereas those with dietary treatment for diabetes and fasting blood glucose (FBG) < 7.5 mmol/L were examined by OGTT. A 75-g OGTT was conducted in the morning (before 11 a.m.), and glucose oxidase technique was performed to measured fasting- and 2-h postload capillary blood glucose values. Participants were fast overnight, avoid heavy physical activity the day before, and avoid smoking the morning prior to the test. According to World Health Organization (WHO) criteria (World Health Organization, 1999), T2D was defined as FBG ≥6.1 (≥110 mg/dl) and/or ≥11.1 mmol/L (≥200 mg/dl) 2 h after glucose load measure on two occasions. The use of drugs, especially metformin, has been implicated in alterations of gut microbiota and microbial products (Mueller et al., 2021). Hence, patients with T2D were excluded if they were under insulin or oral anti-diabetic treatment, such as metformin and sulphonylurea. Furthermore, subjects who reported a cancer diagnosis, chronic inflammatory disease, serious mental disorder, other serious illness, or drug addictions were excluded. Ultimately, the metagenomic sequence datasets for 72 individuals were downloaded with the accession codes, including individuals with NGT (*n* = 43), and untreated patients suffered T2D (T2D; *n* = 29).

### Gene prediction and taxonomic annotation of metagenomic data

The fecal samples were obtained from each participant, and transferred to the laboratory after the screening examination. Samples were stored at –80°C until DNA extraction. Metagenomic DNA was extracted from faecal samples using a standard procedure (Salonen et al., 2010). The concentration of DNA was measured with a Nanodrop instrument (Thermo Scientific, Waltham, Massachusetts, United States) and DNA quality was evaluated by agarose gel electrophoresis. All the isolated DNA samples subjected to sequencing in the Illumina HiSeq2000 instrument with up to 10 samples pooled in one lane. DNA libraries were prepared with a fragment length of 300 bp. With 100 bp in the forward and reverse directions, paired-end reads were generated. Bioinformatics analysis, including gene catalog construction, prediction of genes, taxonomic annotation, and relative abundance calculation, was performed. The genes and related proteins of the gut bacterial genomes were predicted from the assembled contigs through the MetaGeneMark prokaryotic hidden Markov model (v2.10) (Zhu et al., 2010). The Cluster Database at High Identity with Tolerance (CD-HIT, Version 4.5.8, La Jolla, California, United States) was applied to build a non-redundant gene library. The sequence identity cutoff was 0.95, and the minimum coverage cutoff was 0.9. The realignment of the reads to the gene catalog was carried out with SOAP2, and the parameters applied to determine the gene abundance was–m200 –×400 –s119. Genes with more than two mapped reads were included for further assessment. The abundance information of the genes was obtained *via* calculating the total number of reads and normalizing by the gene length. For evaluating the taxonomic annotation, DIAMOND (Version 0.7.9.58, default parameters except that–k 50-sensitive–e 0.00001, Tübingen, Germany) was used to blast the genes to the NR database. A significant match of genes was assessed using e-values ≤10 × e-value of the top hit, and the retained matches were used to differentiate taxonomy. The taxonomical level and abundance of each annotated gene were determined with the lowest common ancestor-based algorithm implemented with MEGAN (MEtaGenome Analyzer, Tübingen, Germany). The abundance of the taxonomy was defined as summing the abundance of genes matched.

### Bioinformatics identification of the lipopolysaccharide-biosynthesis-related enzymes and gut microbiota harboring enzymatic genes

The protein sequences of enzymes participating in LPS biosynthesis were acquired from the RefSeq database of the National Center for Biotechnology Information database (http://www.ncbi.nlm.nih.gov/) (Clementz et al., 1997; Raetz and Whitfield, 2002; Doerrler, 2006), including UDP-N-acetylglucosamine acyltransferase (LpxA), UDP-3-O-[3-hydroxymyristoyl] N-acetylglucosamine deacetylase (LpxC), UDP-3-O-[3-hydroxymyristoyl] glucosamine N-acyltransferase (LpxD), UDP-2,3-diacylglucosamine hydrolase (LpxH), lipid-A-disaccharide synthase (LpxB), tetraacyldisaccharide 4′-kinase (LpxK), 3-deoxy-D-manno-octulosonic-acid transferase (WaaA), Kdo2-lipid IVA lauroyltransferase/acyltransferase (LpxL), and lauroyl-Kdo2-lipid IVA myristoyltransferase (LpxM). BLASTP (version 2.6.0, Bethesda, Maryland, United States) was used with parameters of e-value <1e-5, sequence identity >50%, and coverage >50% as the cutoff in the reference genome resources to identify and align the protein sequences of LPS-biosynthesis-related enzymes within the non-redundant gene sequences of the study cohort. The relative abundance of genes encoding LPS-biosynthesis-related enzymes in each sample was calculated by summing the abundance of non-redundant genes annotated to the same enzyme. The taxonomic classification of gut microbiota harboring enzymatic genes were identified based on the taxonomic annotation of related genes.

### Analysis of lipopolysaccharide-biosynthesis-related kyoto encyclopedia of genes and genomes orthologs

DIAMOND (version 0.7.9.58, Tübingen, Germany) with default parameters, with the exception of–k50 –sensitive–e 0.00001, was applied to align all the genes in the catalog to the Kyoto Encyclopedia of Genes and Genomes (KEGG) database (Release 73.1, Kyoto, Japan). KEGG orthologs (KOs) within the KEGG database platform (https://www.kegg.jp) mainly included target metabolism, genetic information processing, and so forth. Each protein was assigned to a corresponding KO with the highest-scoring annotated hits including ≥1 high-scoring segment pair scoring >60 hits. The query of LPS-biosynthesis-related KOs was performed in the KEGG database using the keywords “Kdo2-lipid A,” “CMP-Kdo,” “UDP-2,3-diacylglucosamine,” “UDP-GlcNAc,” “LPS (lipopolysaccharide),” and enzymes. The sum abundance of all the genes assigned to the identical property was considered as the abundance of a KO. To identify KOs associated with LPS biosynthesis in European women with T2D or NGT, the relative abundance of each KO within the group of samples obtained from patients with T2D was compared with its abundance in individuals with NGT. For each KO, for example, k, the odds ratio (OR) was calculated as follows: OR (k) = [Σs = T2D Ask/Σs = T2D (Σi≠k Asi)]/[Σs = NGT Ask/Σs = NGT (Σi≠k Asi)] as other investigators described previously (Greenblum et al., 2012). Ask denotes the abundance of KO k in sample s, T2D denotes the set of T2D samples, and NGT denotes the group of NGT samples. The structure of the formula could be summarized as [sum(s)/sum(a)], where “sum(s)” denotes the sum of KO1 in the T2D group, divide by sum(a) refers to the sum of non-KO1 in the T2D group. In the present study, the differential abundance score was defined as log2 (OR). Furthermore, the KOs related to LPS-biosynthesis-related enzymes were further classified as T2D-enriched [log2 (OR) > 0] or NGT-enriched [log2 (OR) < 0].

### Statistical analysis

The quantitative variables with normal distribution were expressed as mean ± standard deviation, and the difference between groups was compared using a two-sided *t*-test. Data with non-normal distribution, including KOs, enzymatic genes, and harboring species, were expressed as median and quartiles, and the disparity between NGT and T2D was assessed using the Wilcoxon rank-sum test with Benjamin and Hochberg correction. A *q* value (corrected *p* < 0.05) represented a statistically significant difference. Spearman correlation analysis was performed with *R* (Version 3.3.3, Auckland, New Zealand), and Venn and Sankey plots were graphed with the packages UpSetR and fmsb in *R*, respectively.

## Results

### Public metagenomic sequence datasets of participants with normal glucose tolerance and type 2 diabetes

The fecal shotgun metagenomic sequencing data of 70-year-old European women with normal and diabetic glucose control (Karlsson et al., 2013) were included in the present study to assess the gut microbial potentials for generating LPS and identify candidate LPS-producing bacteria in T2D. Age, sex, and geographical locations have been demonstrated to be confounding factors in investigations regarding the association of gut microbiota with T2D. Hence, the European cohort with elderly women, in whom the incidence of T2D was relatively high, was chosen to minimize the sources of variation. In addition, although Karlsson et al. (2013) suggested that medications in this cohort were unlikely to exert major confounding impacts in the mathematical model based on metagenomic profiles to discriminate patients with T2D, accumulating evidence indicated that the medicine use might directly affect the composition and functions of gut microbiota (Klünemann et al., 2021), especially for metformin (Ma et al., 2021; Mueller et al., 2021). Thus, patients with T2D under drug therapy were excluded from the present study, and ultimately 72 European women with normal (NGT, *n* = 43) or diabetic (T2D, *n* = 29) glucose control were examined.

Biometric and plasma measurements for the 72 participants are shown in Supplementary Table S1. The average age was 70.3 and 70.6 years for NGT individuals and patients with T2D, respectively. Glucose-related factors, such as fasting blood glucose, fasting insulin, and HbA1c, were significantly higher in patients with T2D (all *p* < 0.001). In addition, BMI, WC, and leptin and C-peptide levels were enriched among patients with T2D. TC and adiponectin levels significantly increased in the control group, while other baseline characteristics showed no difference between the two groups. The metagenomic data of genomic DNA from 72 fecal samples based on Illumina HiSeq 2000 were 217 Gb paired-end reads, with an average of 3.0 ± 1.2 Gb (mean ± SD) for each sample.

### Enzymatic genes for lipopolysaccharide biosynthesis in the gut of patients with type 2 diabetes

**FIGURE 1:**
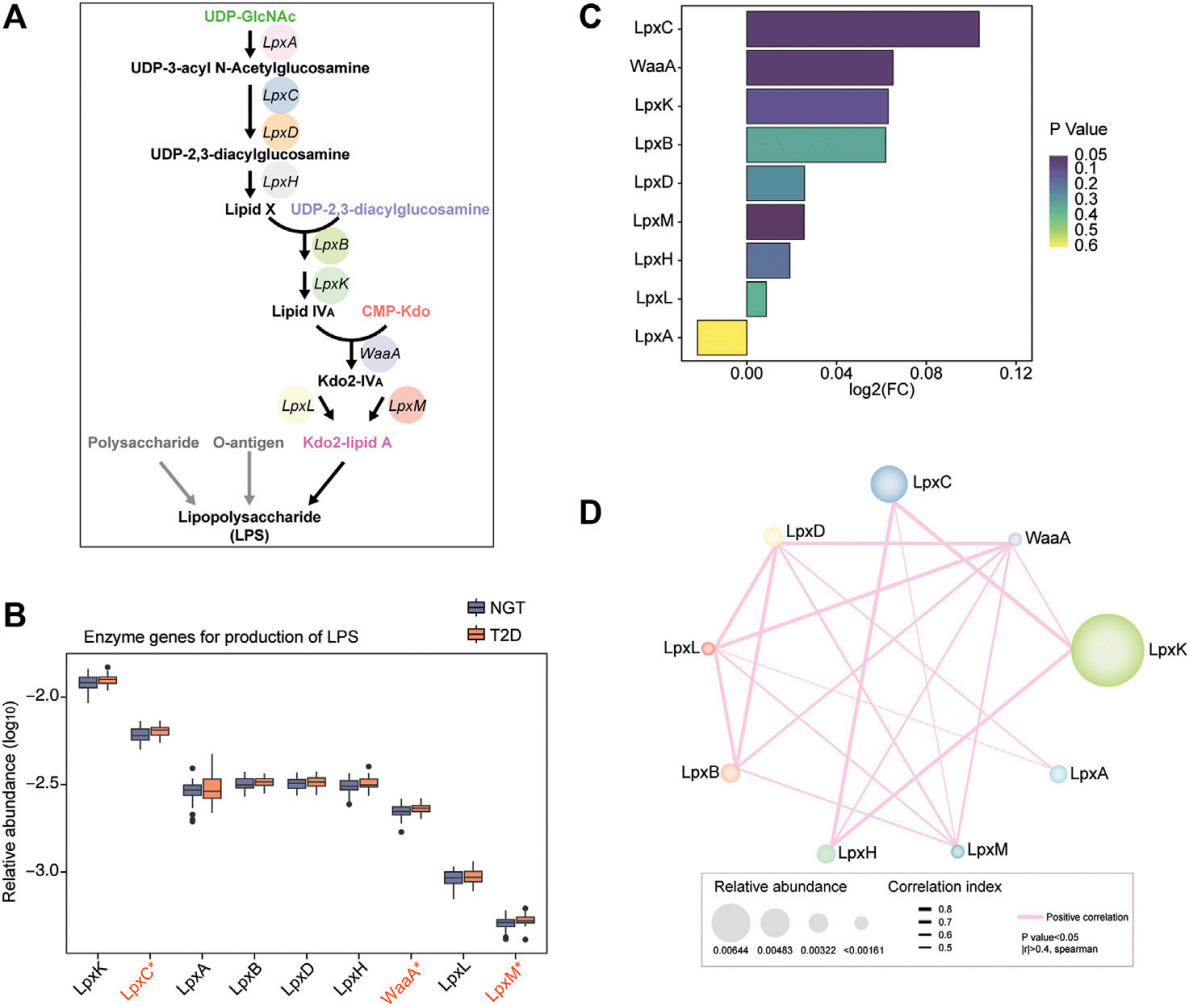
Relative abundance of LPS-biosynthesis-related enzymes in the gut of individuals with NGT and patients with T2D. **(A)** Schematic diagram of the direct synthetic process and transformation pathways for LPS biosynthesis. Key enzymes catalyzing the progress from UDP-GlcNAc to Kdo2-lipid A were crucial and necessary for LPS biosynthesis, and genes encoding these enzymes were targeted for exploring their abundance. LpxA, UDP-N-acetylglucosamine acyltransferase; LpxC, UDP-3-O-[3-hydroxymyristoyl] N-acetylglucosamine deacetylase; LpxD, UDP-3-O-[3-hydroxymyristoyl] glucosamine N-acyltransferase; LpxH, UDP-2,3-diacylglucosamine hydrolase; LpxB, lipid-A-disaccharide synthase; LpxK, tetraacyldisaccharide 4′-kinase; WaaA, 3-deoxy-D-manno-octulosonic-acid transferase; LpxL, Kdo2-lipid IVA lauroyltransferase/acyltransferase; LpxM, lauroyl-Kdo2-lipid IVA myristoyltransferase. **(B)** Box plots depicting the relative abundance levels of LPS-biosynthesis-related enzymes between individuals with NGT and patients with T2D based on the genes encoding the enzymes (orange, T2D; blue, NGT). Boxes represent the interquartile ranges; the inside lines indicate the median; circles are outliers, and red asterisk represents statistical difference at *p* < 0.05 (Wilcoxon rank-sum test). **(C)** Enrichment of LPS-producing enzymatic genes according to the encoding genes was evaluated using Log2 (fold change T2D/NGT, FC). Log2 (FC) > 0 and Log2 (FC) < 0 indicate the enrichment of enzymes in patients with T2D and individuals with NGT, respectively. The colors from dark purple to light yellow indicate the scales of *p*-value. **(D)** Co-occurrence network of the LPS-biosynthesis-related enzymes in the study cohort. The numbers labeled in the enzymes indicate the number of connections with others. The thresholds calculated *via* Spearman’s correlation analyses were *p*-value < 0.05 and |*r*| >0.4. The pink lines connecting two enzymes denote a positive correlation. Line thickness and node size represent the |*r*| value of correlation and relative abundance of enzymatic genes, respectively. T2D, type 2 diabetes; NGT, normal glucose tolerance.

LPS is constituted of polysaccharide, O-antigen, and Kdo2-lipid A, and the crucial step of LPS biosynthesis is the process of Kdo2-lipid A biosynthesis. As the key component of LPS, Kdo2-lipid A is known to be catalyzed from UDP-GlcNAc, progressively to UDP-3-acyl N-acetylglucosamine, UDP-2,3-diacylglucosamine, Lipid X, Lipid IVA, and Kdo2-IVA, by using nine synthetic enzymes, including LpxA, LpxC, LpxD, LpxH, LpxB, LpxK, WaaA, LpxL, and LpxM (Figure 1A). These enzymes were considered to be responsible for LPS biosynthesis. We thus investigated their enrichment in the gut of individuals with NGT and patients with T2D based on the encoding gene sequence. LpxK and LpxC were observed to be the most abundant enzymes in the study cohort, while the level of LpxL and LpxM were relatively low (Figure 1B). According to the relative abundance of the total nine enzymes in each individual, PCA scatter plots roughly distinguished samples from individuals with NGT and patients with T2D, and a significant disparity between the groups was obtained in a single coordinate axis at PCA1 (Supplementary Figure S1).

The abundance of microbial genes for LPS-biosynthesis-related enzymes was mostly enhanced in the gut of patients with T2D, except for LpxA, compared with NGT controls. Intriguingly, the enzymatic gene abundance for LpxC, WaaA, and LpxM was strikingly augmented in patients with T2D, and LpxC coupled with WaaA displayed a higher fold change in T2D *versus* NGT (Figures 1B,C). The correlation and co-abundance relationship between LPS- biosynthesis-related enzymes in the study cohort were examined, and it was detected that LpxC was directly linked to LpxK, LpxH, and LpxM (Figure 1D). However, WaaA closely related to almost all the other enzymes, especially LpxD, LpxL, LpxB, LpxH, LpxM, and LpxK. The coordinated alterations of LPS-producing enzymes were confirmed, and this information suggested that the capacity and potential for LPS biosynthesis in the gut of patients subjected to T2D might be extremely elevated.

Other gut microbial metabolic products well established to participate in host physiology and pathology, including TMAO and SCFA derived from dietary choline, carnitine, phosphatidylcholines, and dietary fiber, were also focused. Although yeaX and CntA showed a reduced tendency, we found that the enzymatic genes for the biosynthesis of trimethylamine (TMA) were non-discriminatory between individuals with NGT and patients with T2D, as shown by CutD, CutC, TorA, CntB, GrdH, yeaX, and CntA in Supplementary Figures S2A,B. For enzymatic genes essential for SCFA biosynthesis, most enzymes increased in patients with T2D, such as propionyl CoA transferase, yciA and tesB, and CO dehydrogenase acetyl CoA synthase complex as well as tesA (Supplementary Figures S2C,D).

### Intestinal functions related to lipopolysaccharide biosynthesis in European women with type 2 diabetes

**FIGURE 2:**
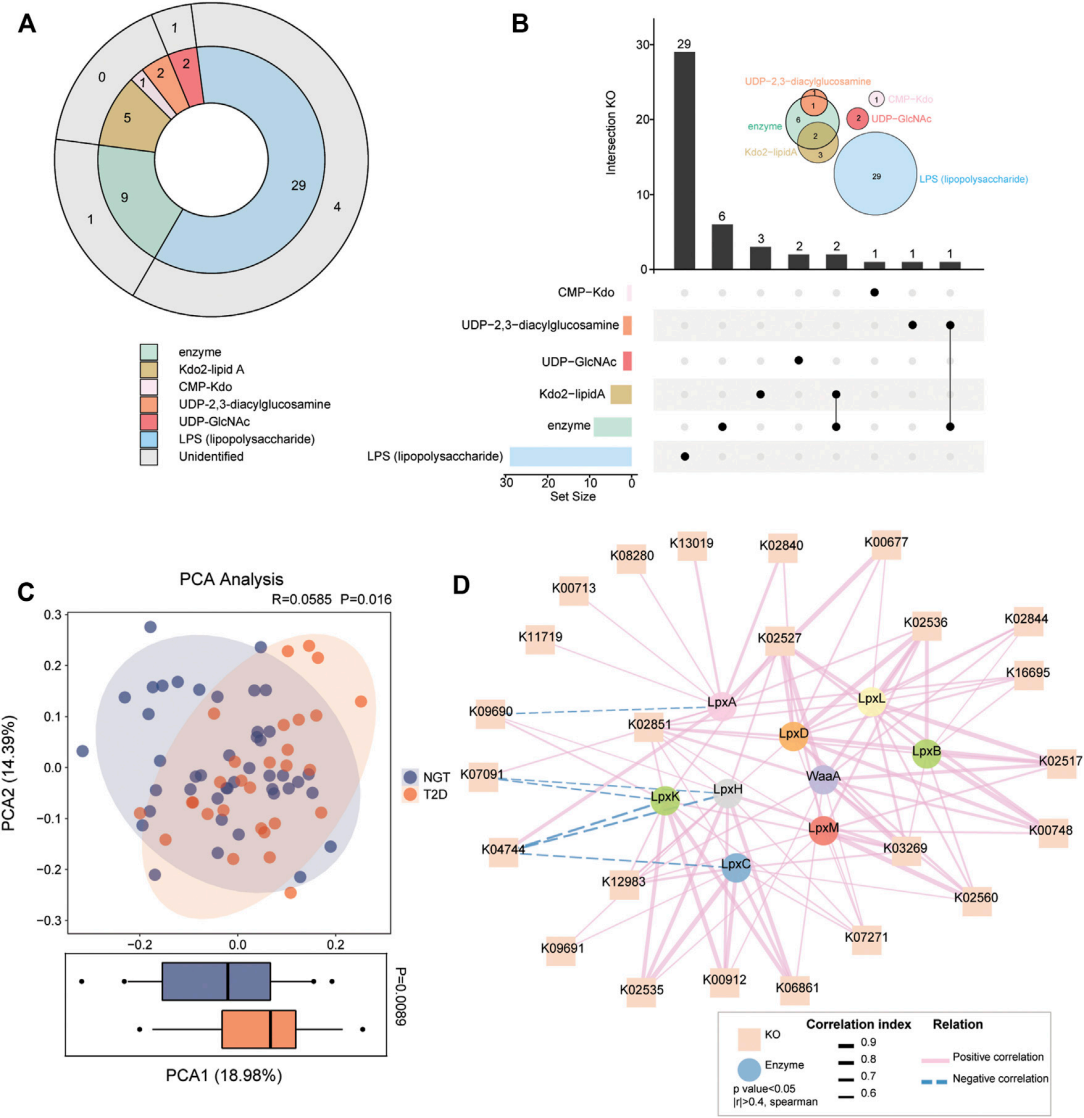
Gut microbial KOs implicated in LPS biosynthesis in individuals with NGT and patients with T2D. **(A)** KOs involved in synthetic progress and transformation pathways for LPS biosynthesis were identified through related compounds and enzymes. Different compounds or enzymes are shown in different colors. The outer circle in gray indicates the six KOs not detected in the present cohort. **(B)** UpSet plot and Venn diagram analysis showed the coexisting KO distributions in LPS-biosynthesis-related compounds and enzymes. The bar plot at the bottom left shows the number of KOs implicated in each compound and enzyme. The dot and line at the bottom right represent the unique and shared KO intersection by different compounds and enzymes, respectively. The number of specific KOs and shared KO intersections are shown in the black histogram above. Venn diagrams at the upper right show the number of annotated KOs involved in LPS-biosynthesis-related compounds and enzymes. One KO was shared by the LPS-biosynthesis-related enzymes and UDP-2,3-diacylglycosamine, and two KOs are shared by Kdo2-lipidA and enzymes. **(C)** PCA analysis based on the 45 annotated KOs across individuals with NGT and patients with T2D. *p* = 0.016 between groups, ANOSIM test. The distribution of patients with T2D according to KOs was significantly different from that in NGT at the PCA1 axis (*p* = 0.0089, Wilcoxon rank-sum test). **(D)** Correlation network reflecting the interaction between LPS-biosynthesis-related enzymes (circle) and KOs (square). Spearmen correlation analysis, |*r*| >0.4, *p* < 0.05. Lines connecting nodes indicate positive (solid lines in red) or negative (dotted lines in blue) correlations, and the thickness of lines is proportional to the correlation coefficients. T2D, type 2 diabetes; NGT, normal glucose tolerance.

Considering the shifts of enzymatic genes for LPS formation in patients with T2D, the gut microbial functions relevant to the synthetic process and transformation pathways of LPS biosynthesis were assessed. Overall, 29 KOs were annotated with LPS, 2 KOs with UDP-GlcNAc, 2 KOs with UDP-2,3-diacylglucosamine, 1 KO with CMP-Kdo, 5 KOs with CMP-Kdo, and 9 KOs with Kdo2-lipid A-related enzymes through the KEGG database in the present study cohort, while unidentified KOs in NGT and T2D were not followed up (Figure 2A). We found 2 enzyme-associated KOs shared with Kdo2-lipidA, 1 KO coexisting with UDP-2,3-diacylglucosamine, and 45 KOs specific for LPS-biosynthesis-related compounds (Figure 2B). We clearly separated patients with T2D from individuals with NGT *via* PCA based on the relative abundance of the aforementioned 45 KOs in the gut (Figure 2C). In addition, an obvious distinction at component 1, which could explain 18.98% of the variation between NGTs and T2Ds, was detected. We thus performed Spearmen correlation analysis to investigate the linkage between 9 LPS-biosynthesis-related enzymes and 45 KOs (Figure 2D). Among the 45 annotated KOs, we identified 24 KOs prominently associated with all the enzymes. Nine KOs were positively correlated with LpxC, and one KO was negatively correlated with it. A total of 12 positive correlations were found between KOs and WaaA. KOs such as K02535 and K03269 were positively correlated with LpxM.

**FIGURE 3:**
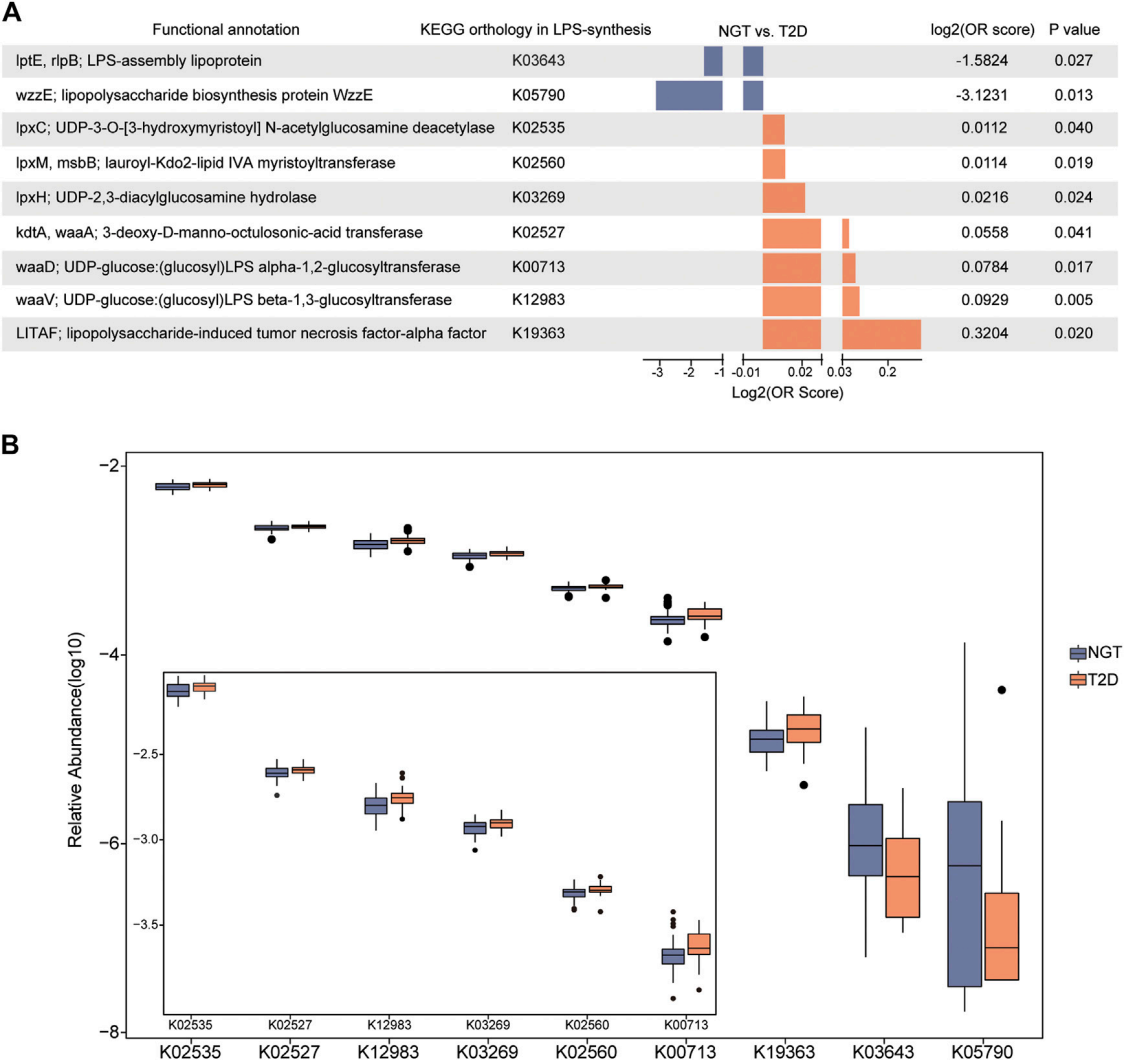
Disparate abundance of gut microbial KOs associated with LPS biosynthesis between individuals with NGT and patients with T2D. **(A)** Enrichment of the LPS-biosynthesis-related KOs with distinct abundance between individuals with NGT and patients with T2D. Log2 (OR score) < 0 (enriched in NGT, blue); Log2 (OR score) > 0 (enriched in T2D, orange), *p* < 0.05. **(B)** Box plots illustrating the relative abundance of KOs varied significantly between the NGT and T2D groups. Horizontal lines within the box plots represent median values, upper and lower ranges of the box represent 75% and 25% quartiles, respectively, and circles represent outliers. T2D, type 2 diabetes; NGT, normal glucose tolerance.

A comparison of KO enrichment between NGTs and T2Ds demonstrated nine differently distributed KOs, including K03643 (LPS-assembly lipoprotein), K05790 (lipopolysaccharide biosynthesis protein), K02535 (UDP-3-O-[3-hydroxymyristoyl] N-acetylglucosamine deacetylase), K02560 (lauroyl-Kdo2-lipid IVA myristoyltransferase), K03269 (UDP-2,3-diacylglucosamine hydrolase), K02527 (3-deoxy-D-manno-octulosonic-acid transferase), K00713 (LPS alpha-1,2-glucosyltransferase), K12983 (LPS beta-1,3-glucosyltransferase), and K19363 (lipopolysaccharide-induced tumor necrosis factor-alpha factor) (Figures 3A,B). Seven potential LPS-biosynthesis functional orthologs of K02535, K02560, K03269, K02527, K00713, K12983, and K19363 remarkably increased in the gut communities of patients with T2D compared with the control participants, while K03643 and K05790 were more abundant in NGTs. These findings further suggested aggravated microbial functions toward the formation of LPS in patients with T2D.

### Candidate lipopolysaccharide-producing gut microbiota containing biosynthesis-related enzymatic genes

**FIGURE 4:**
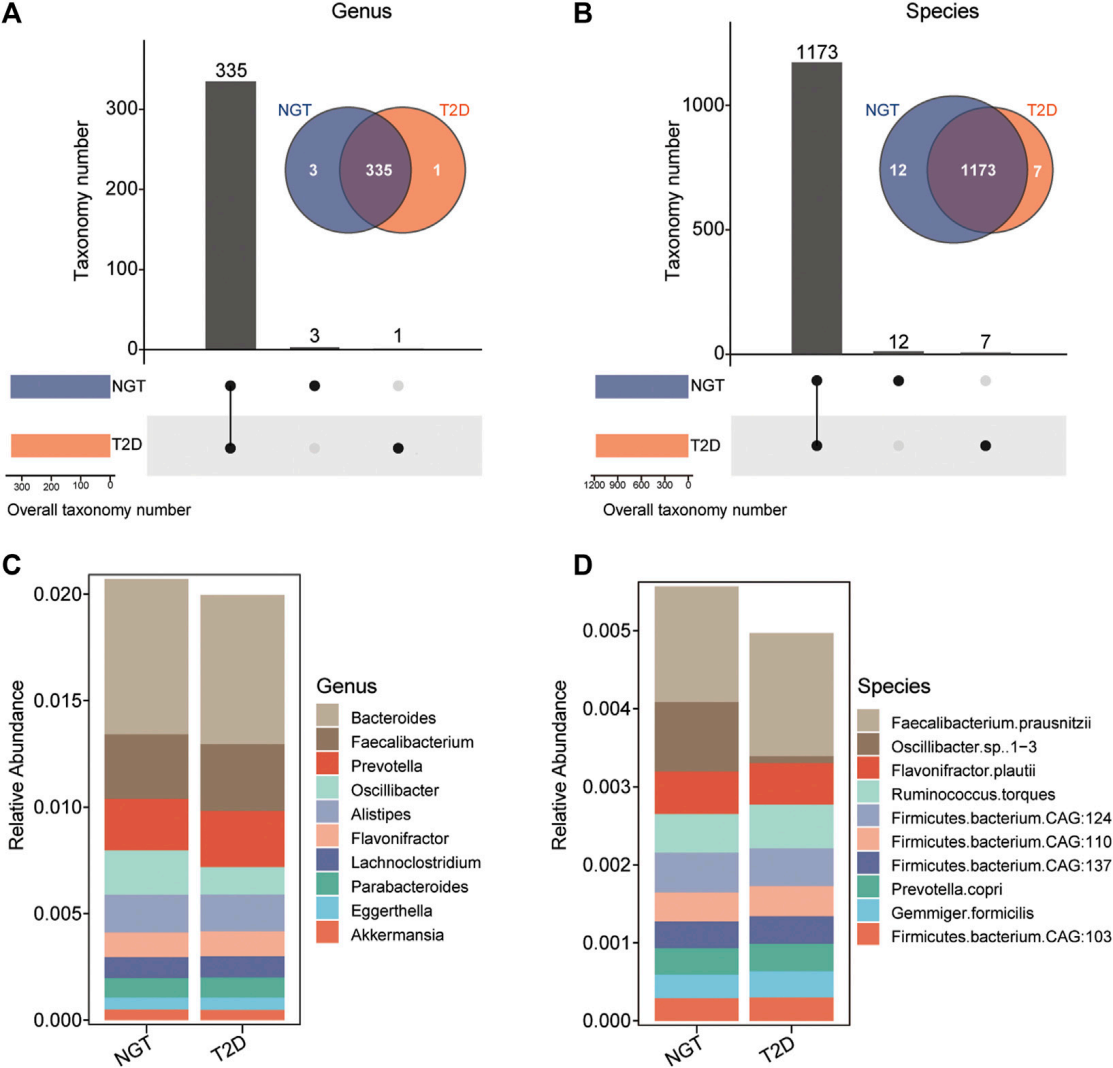
Taxonomic identification and the distribution of bacteria harboring LPS-biosynthesis-related enzymatic genes in individuals with NGT and patients with T2D. **(A,B)** UpSet plot and Venn diagram showing the distribution of bacteria harboring LPS-biosynthesis-related enzymatic genes, shared and unique in the NGT and T2D groups at the genus, and species levels, respectively. Bar charts above represent the number of genera, and species harboring LPS-biosynthesis-related enzymatic genes, shared and specific between groups. At the bottom left, bar charts indicate the bacteria that harbored LPS-biosynthesis-related enzymatic genes in each group (orange, T2D; blue, NGT). The line between nodes at the bottom right denotes the intersected taxon within each group. **(C,D)** Stack columns depicting the relative abundance for the top 10 genera and species with LPS-producing potentials (enzymatic genes) in NGT and T2D. T2D, type 2 diabetes; NGT, normal glucose tolerance.

The enzymatic genes of LpxA, LpxC, LpxD, LpxH, LpxB, LpxK, WaaA, LpxL, and LpxM were aligned to the integrated nr database to identify the gut microbiota in which LPS-biosynthesis-related enzymes existed. The enzymatic distribution together with the taxonomic identification of enzyme-harboring bacteria was evaluated in individuals with NGT and patients with T2D (Supplementary Figure S3). Precisely, LpxC existed in 162 genera, and 536 species; LpxM distributed in 42 genera, and 89 species; 107 genera, and 316 species carried WaaA genes. In addition, the most abundant enzyme LpxK was detected in 229 genera, and 815 species. By removing the overlapped taxonomy between distinct enzymes, 339 unduplicated genera, and 1,192 non-repetitive species were identified in the study cohort (Figures 4A,B).

**FIGURE 5:**
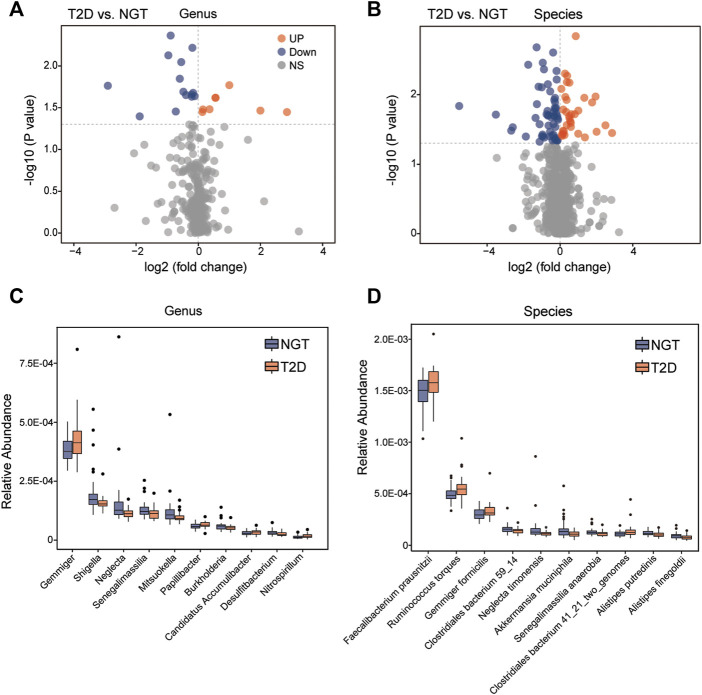
Abundance comparisons of candidate LPS-producing bacteria across the NGT and T2D groups. **(A,B)** Twenty-two genera, and 89 species harboring LPS-biosynthesis-related enzymatic genes were identified to be significantly different between individuals with NGT and patients with T2D using volcano plots, respectively. Orange and blue indicate significant enrichment and reduction in the T2D group, respectively; gray denotes a non-significant difference between the two groups. **(C,D)** Box plots showing the relative abundance of the top 10 gut genera and species with both genes for LPS-biosynthesis-related enzymes, and statistical difference between the NGT and T2D groups. Boxes represent the interquartile ranges, the inside lines represent the median, and circles represent outliers. Orange and blue represent enrichment in patients with T2D and individuals with NGT, respectively. T2D, type 2 diabetes; NGT, normal glucose tolerance.

As a result, 335 genera, and 1,173 species harboring at least 1 LPS-biosynthesis-related enzymes were detectable in both individuals with NGT and patients with T2D. The top species related to LPS-producing enzymes were *Faecalibacterium prausnitzii* from Faecalibacterium, and *Flavonifractor plautii* from Flavonifractor (Figures 4C,D). Among these gut microbiota with LPS-biosynthesis-related enzymatic genes, we found 8 genera, and 33 species prominently enhanced in patients with T2D, while the abundance levels of 14 genera, and 56 species markedly reduced in the gut of patients with T2D (Figures 5A,B). For instance, *Gemmiger, Papillibacter*, *Nitrospirillum*, *F. prausnitzii*, *Ruminococcus torques*, and *Gemmiger formicilis* were remarkably enriched in patients with T2D, while those diminished in T2D mainly belonged to *Shigella, Neglecta*, *Senegalimassilia*, and *Neglecta timonensis*. (Figures 5C,D).

### Correlation of lipopolysaccharide-biosynthesis-related enzymatic genes with gut microbiota and functional activities

**FIGURE 6:**
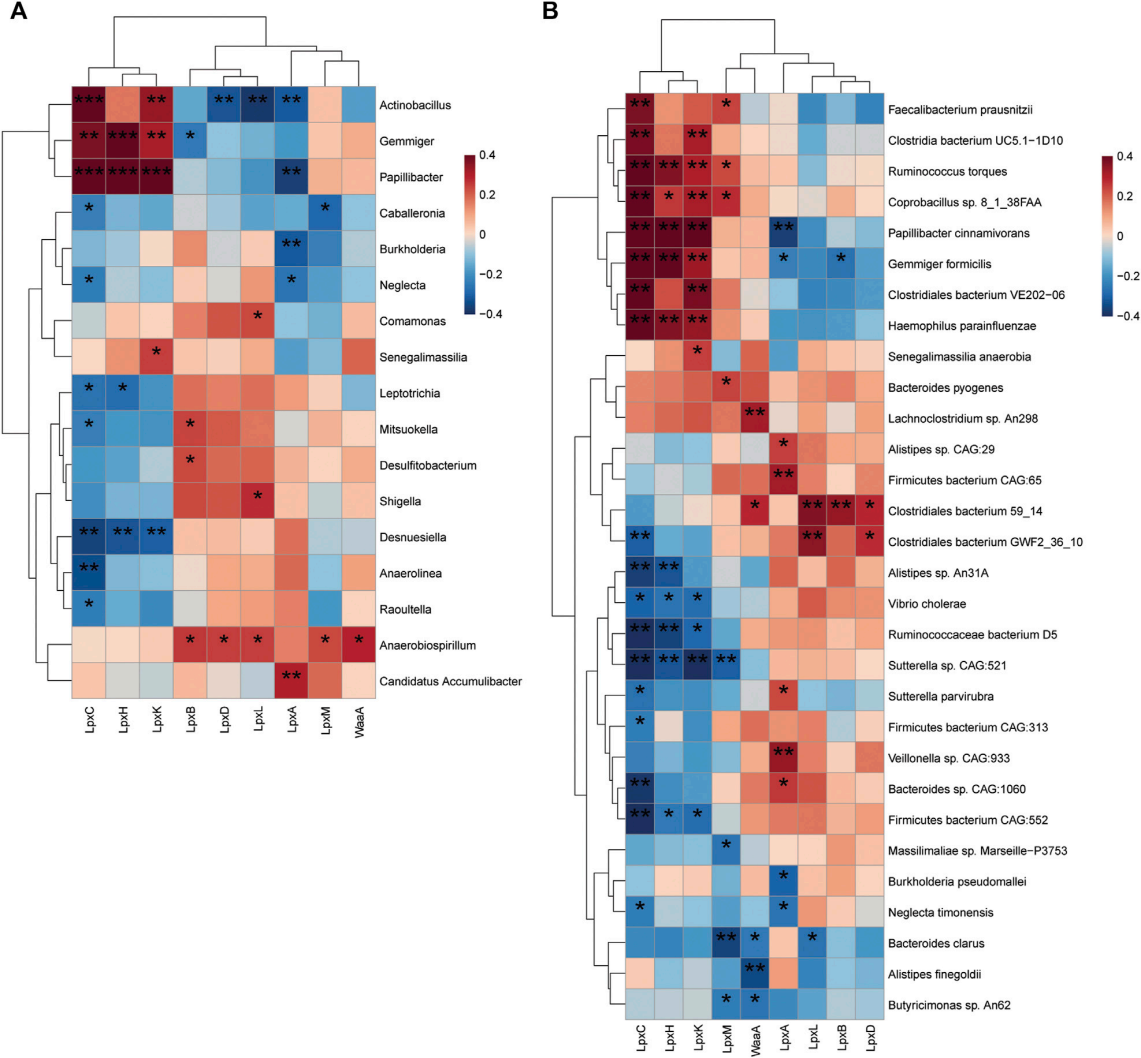
Correlation of LPS-biosynthesis-related enzymes and harboring gut bacteria. **(A)** Spearmen correlation analysis of the relationship between potential LPS-producing genera (22 bacteria discriminative in patients with T2D and individuals with NGT) and enzymatic genes. Statistical differences in correlation coefficients between LPS-producing genus and one of the enzyme genes were shown **(B)** Heat map depicts the association between LPS-biosynthesis-related enzymes and top 30 altered species harboring enzymatic genes. Blue, negative correlation; red, positive correlation; ^*^ *p* < 0.05; ^**^ *p* < 0.01; ^***^ *p* < 0.001. Correlation with *p* ≥ 0.05 is not shown. T2D, type 2 diabetes; NGT, normal glucose tolerance.

The interactions between the enzymatic genes of LpxA, LpxC, LpxD, LpxH, LpxB, LpxK, WaaA, LpxL, and LpxM and discriminative taxonomy in patients with T2D and individuals with NGT were examined to further explore the key LPS-producing microbes in patients with T2D. At the genus level, a positive correlation was detected between the abundance of *Actinobacillus, Gemmiger*, *Papillibacter*, and enzyme LpxC (Figure 6A). The abundance of Anaerobiospirillum showed a positive correlation simultaneously with LpxB, LpxD, LpxL, LpxM and WaaA. We also found that the abundance of intestinal species, including *R. torques*, *Papillibacter cinnamivorans*, *Bacteroides pyogenes* and *Lanchnoclostridium* sp., correlated with higher levels of LpxC, LpxM, and WaaA, respectively (Figure 6B).

**FIGURE 7:**
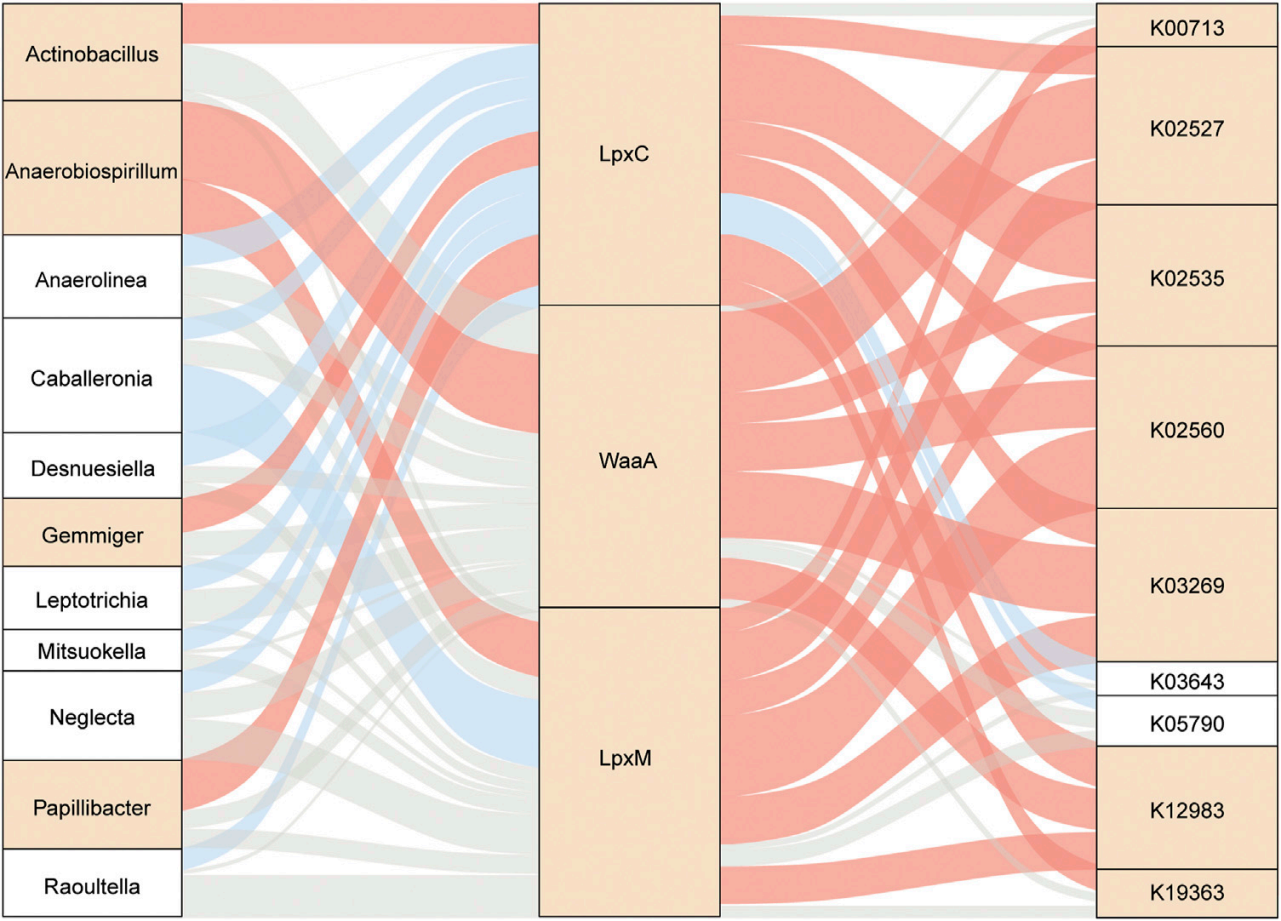
Interrelationship across gut microbes, LPS-biosynthesis-related enzymatic genes, and respective KOs. Sankey diagram based on Spearman correlation analysis. The gut microbes with the potential to produce LPS were related to LPS-biosynthesis-related enzymes and further linked to microbial KOs during LPS biosynthesis. Red, positive correlation (*p* < 0.05); blue, negative correlation (*p* < 0.05); gray, non-significant correlation (*p* ≥ 0.05). Square in yellow, enriched in T2D; square in white, enriched in NGT. T2D, type 2 diabetes; NGT, normal glucose tolerance.

Taken together, further analysis of the relationship among enzyme-harboring bacteria, LpxC, LpxM, WaaA, and LPS-biosynthesis-related KOs revealed 11 gut genera directly linked to the enzymes and subsequently associated with the 9 LPS-biosynthesis functional orthologs to varying degrees (Figure 7). The complicated correlation and network indicated that the overgrowth in potential LPS-producers, including *Actinobacillus, Anaerobiospirillum, Gemmiger*, and *Papillibacter*, might be responsible for the enhancement of LPS-biosynthesis-related enzymes in the gut of patients with T2D, and thus might potentially contribute to the excessive activation of bacterial functions implicated in LPS formation, such as LPS alpha-1,2-glucosyltransferase, 3-deoxy-D-manno-octulosonic-acid transferase, UDP-3-O-[3-hydroxymyristoyl] N-acetylglucosamine deacetylase, lauroyl-Kdo2-lipid IVA myristoyltransferase, UDP-2,3-diacylglucosamine hydrolase, and LPS beta-1,3-glucosyltransferase.

## Discussion

In the present study, we profiled the gut microbial enzymes with potentials in LPS biosynthesis and acquired indications demonstrating LPS biosynthesis pathways, as well as potential bacterial producers of LPS in European women with DM based on a metagenomic shotgun sequencing analysis. The major findings of this study included as follows: 1) LPS-biosynthesis-related enzymes roughly distinguished individuals with NGT from patients with T2D. Specifically, almost the levels of all the LPS-biosynthesis-related enzymes were enhanced in the gut of patients with T2D; especially the levels of LpxC, WaaA, and LpxM were significantly higher in patients with T2D. 2) By aligning to the integrated NR database, 339 unduplicated genera, and 1,192 non-repetitive species were identified as bacteria harboring LPS-biosynthesis-related enzymatic genes. 3) Among these gut microbiota containing LPS-biosynthesis-related enzymatic genes, we found that genera Gemmiger and Paoillibacter, and species *R. torques* and *G. formicilis* were prominently enriched in patients with T2D. 4) Furthermore, the correlation analysis showed that gut genera were potentially involved in the development of T2D by linking to LPS enzymes (LpxC, WaaA, and LpxM) and subsequently associated with LPS-biosynthesis-related KOs.

The highly prevalent DM is crucial on the international health agenda as a global epidemic posing a threat to human health and global economy (Zimmet et al., 2014). Previous investigations documented that the prevalence of DM during 2002–2007 augmented significantly by 44% in men and 17% in women in Sweden compared with the prevalence in 1990–1995 (Lindahl et al., 2010). In particular, among women, the morbidity of DM was twice in 50-year-olds compared with 40-year-olds, and quadruple in 60-year-olds compared with 40-year-olds (Lindahl et al., 2010). Therefore, elderly female patients with T2D attracted more attention. We implemented the present findings to characterize microbial features and profiles within the gastrointestinal tract of these populations by mining previous metagenomics databases.

It has been widely recognized that multiple metabolites derived from the gut microbiota might play vital roles in the pathogenesis and development of metabolic disease such as DM (Burcelin, 2016). More importantly, LPS, as a notable gut microbial metabolite, was frequently linked with various human diseases. Loeser et al. (2022) revealed that patients with obesity-associated osteoarthritis exhibited extremely higher serum levels of LPS compared with healthy populations. In addition, Zhao et al. (2017) reported the presence of bacterial LPS within brain lysates from the hippocampus and superior temporal lobe neocortex in the brains of patients with Alzheimer’s disease. It is important to note that emerging studies have provided supportive evidence for the essential role of LPS in insulin resistance. LPS was demonstrated to activate a status of chronic low-grade inflammation by interacting with type 4 Toll-like receptors (Kayagaki et al., 2013; Salazar et al., 2020), which mainly presented in monocytes, favoring the release of pro-inflammatory cytokines and ultimately enabling an insulin resistance state in the later stages (Khondkaryan et al., 2018; Huang et al., 2019). Further, the treatment with antibiotics to mice fed a high-fat diet reduced metabolic endotoxemia and decreased the content of LPS in the cecum. These effects were suggested to be associated with suppressed glucose intolerance, alleviative inflammation, and oxidative stress (Cani et al., 2008). Uncovering the LPS-biosynthesis-related bacteria and the biosynthesis capabilities of the human gut microbiota might have significance in identifying the mechanisms underlying the development of T2D.

In studies on humans, accumulating evidence indicated that the injection with LPS could elicit a systemic inflammatory response with enhanced neutrophil counts, increased temperature, and heart rate, as well as augmented plasma concentrations of cytokines and biosynthesis of adhesion molecules in volunteers with type 2 diabetes (Andreasen et al., 2010). Recent reports highlighted the finding that female patients with T2D possessed significantly lower LPS levels following the administration of resistant dextrin for 8 weeks (Farhangi et al., 2018). We showed that most of the LPS-biosynthesis-related enzymes in microorganisms were highly expressed within the gastrointestinal tract of patients with T2D, especially for key enzymes as LpxC, WaaA, and LpxM, suggesting an enhanced possibility of LPS biosynthesis in patients with T2D than in the healthy controls. Similarly, enzymatic genes associated with microbial metabolites, such as TMAO (Zuo et al., 2020), SCFA (Zhang J. et al., 2021), and branched-chain amino acids (Zhang et al., 2022), have been previously used to explore critical metabolic enzymes and potential microbial taxa responsible for metabolite biosynthesis in diseases. Microbial trimethylamine (TMA)-lyase-containing species *L. saccharolyticum* was recently validated to effectively produce TMA both *in vivo* and *in vitro* (Cai et al., 2022). Notably, the investigators previously reported that non-typeable *H. influenzae* NTHi, which contained the LPS-biosynthesis-related genes, was regarded as a frequent cause of upper and lower respiratory tract infections such as otitis media and pneumonia (Fox et al., 2005). Therefore, LpxC, WaaA, and LpxM were critical genes in LPS biosynthesis pathways. We proposed that the downregulation of these genes might result in the reduction of LPS biosynthesis in T2D. These studies encouraged us to further explore the relationship of LPS enzyme-containing gut microbiota and LPS-biosynthesis-related pathways with T2D. Studies indicated that the dysbiosis of the gut microbiota might disturb intestinal barrier functions and impact host metabolic and signaling pathways, which were directly or indirectly related to T2D insulin resistance (Sharma and Tripathi, 2019). Yu et al., 2019 showed that the metabolic parameters, such as fasting blood glucose and food intake, were altered significantly when fecal bacteria from db/db mice were transplanted into pseudo-germ-free mice. It was confirmed that abnormal gut microbiota composition might contribute to the onset and occurrence of T2D. Meanwhile, in the present study, some gut microbiota harboring LPS-biosynthesis-related enzymatic genes, such as *Gemmiger, Papillibacter*, which might potentially produce enzymes responsible for the biosynthesis of LPS, were found to be dramatically enriched in female patients with T2D. In addition, several KOs, including LPS-assembly lipoprotein, were prominent in patients with T2D. Thus, it was demonstrated that the gut bacterial genes of enzymes responsible for LPS biosynthesis, potential microbial functions pertaining to LPS biosynthesis, and key LPS-biosynthesis-related gut microbiota were significantly enhanced in patients with T2D, ultimately contributing to the exacerbated accumulation of LPS in T2D.

## Conclusion

In conclusion, we described LPS-biosynthesis profiles in the intestinal tract of European women with NGT or untreated T2D. The key LPS-biosynthesis-related microbial enzymes and pathways and gut microbiota responsible for LPS formation were abundant in patients with T2D, which were consequently linked to abnormal LPS levels. These findings might provide an advanced understanding of the relationship between gut microbiota and T2D. They might also have clinical value in introducing the therapy for T2D by focusing on LPS-biosynthesis-related gut microbiota in the future.

## Data Availability

The data presented in the study are deposited in the Sequence Read Archive repository, accession number ERP002469.
