# Supplementary material for: Potential of gut microbiota for lipopolysaccharide biosynthesis in European women with type 2 diabetes based on metagenome
Source: Front Cell Dev Biol. 2022 Oct 11;10:1027413. doi: 10.3389/fcell.2022.1027413 (PMC9592851; doi:10.3389/fcell.2022.1027413)
Supplement: Supplementary file 1 [file Table1.DOCX]

| Supplementary Table 1. Baseline characteristics of study population | | | |
| --- | --- | --- | --- |
| Variables | NGT (n=43) | T2D (n=29) | P value |
| Age, years | 70.3±0.7 | 70.6±0.7 | 0.124 |
| BMI, kg/m^2^ | 25.8±4.4 | 28.3±5.6 | 0.036 |
| WHR, cm/cm | 0.8±0.1 | 0.9±0.1 | 0.060 |
| WC, cm | 84.1±9.2 | 92.5±12.0 | 0.001 |
| TC, mmol/L | 5.74±0.85 | 5.23±1.16 | 0.035 |
| TG, mmol/L | 0.93 (0.78-1.18) | 1.06 (0.94,1.43) | 0.086 |
| HDLC, mmol/L | 1.96±0.50 | 1.74±0.48 | 0.064 |
| LDLC, mmol/L | 3.30±0.83 | 2.93±1.07 | 0.099 |
| FBG, mmol/L | 5.43±0.65 (n=42) | 6.77±1.18 | <0.001 |
| Creatinine, µmol/L | 69.00 (61.00-74.00) | 65.00 (59.00-72.00) | 0.227 |
| Fasting insulin, mU/L | 6.70 (5.10-9.60) | 12.00 (7.90-16.00) | <0.001 |
| HbA1c, mmol/mol | 36.00 (35.00-38.00) | 43.00 (40.00-45.00) | <0.001 |
| Adiponectin, mg/L | 14.40 (11.70-19.00) | 10.70 (8.91-15.10) | 0.004 |
| Leptin, µg/L | 15.10 (8.53-25.70) | 25.60 (13.10-34.70) | 0.040 |
| GLP-1, pmol/L | 0.80 (0.20-1.40) (n=38) | 1.10 (0.40-1.90) | 0.162 |
| FGF-19, pg/ml | 100.30 (67.19-146.48) (n=38) | 107.27 (76.65-138.55) | 0.591 |
| hsCRP, mg/L | 1.47 (0.83-2.53) | 2.23 (1.37-3.54) | 0.078 |
| C-peptide, nmol/L | 0.64 (0.54-0.81) | 0.99 (0.74-1.21) | <0.001 |
| TNFα, ng/L | 1.84 (1.33-2.62) | 1.88 (1.56-2.71) | 0.466 |
| CD163, ng/ml | 693.16 (592.01-867.17) (n=38) | 763.23 (578.90-889.54) | 0.975 |
