# Supplementary figures and images for: Potential of gut microbiota for lipopolysaccharide biosynthesis in European women with type 2 diabetes based on metagenome
Source: Front Cell Dev Biol. 2022 Oct 11;10:1027413. doi: 10.3389/fcell.2022.1027413 (PMC9592851; doi:10.3389/fcell.2022.1027413)

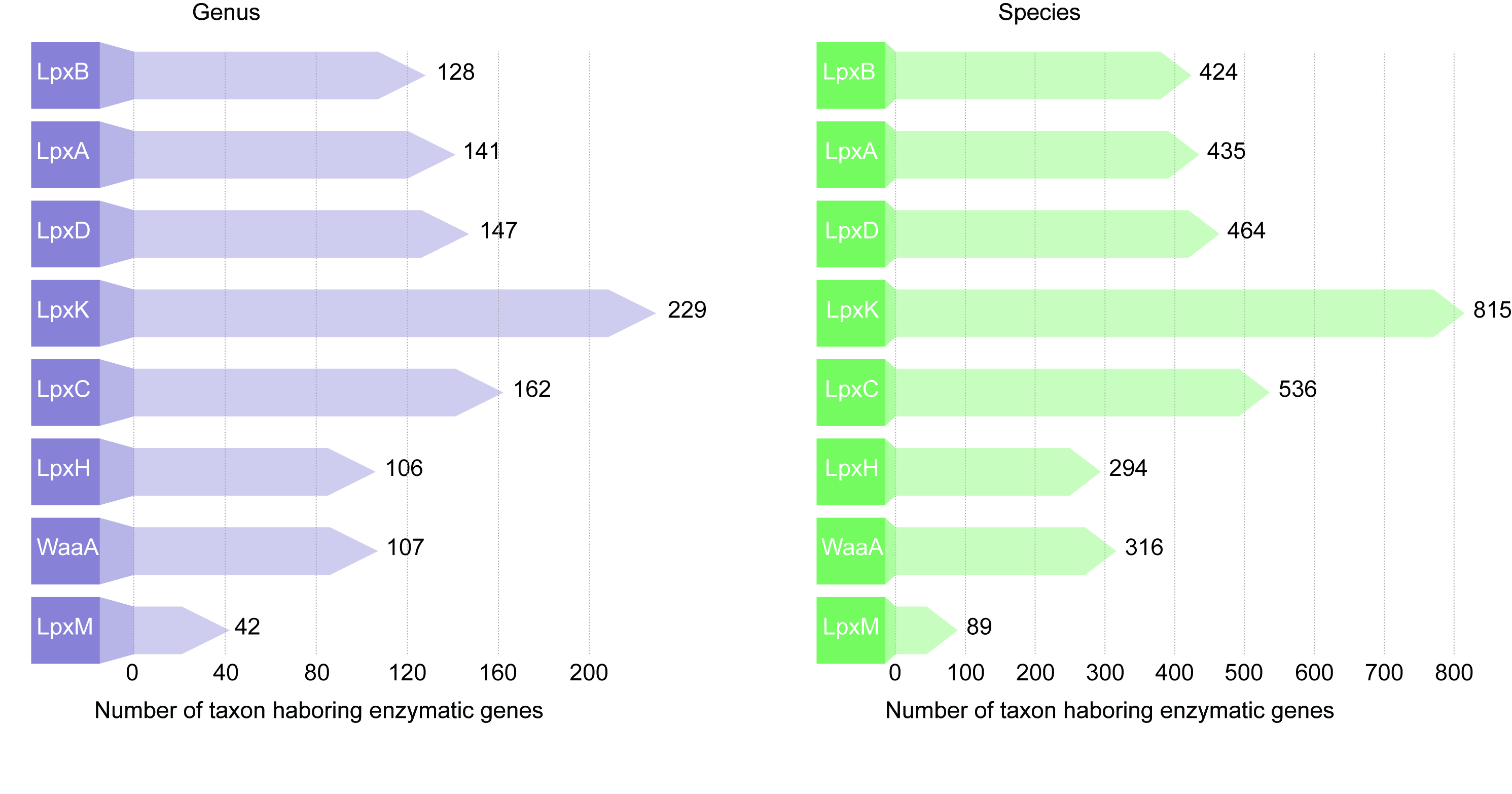

Supplement: Supplementary file 2 [file Image3.TIF]

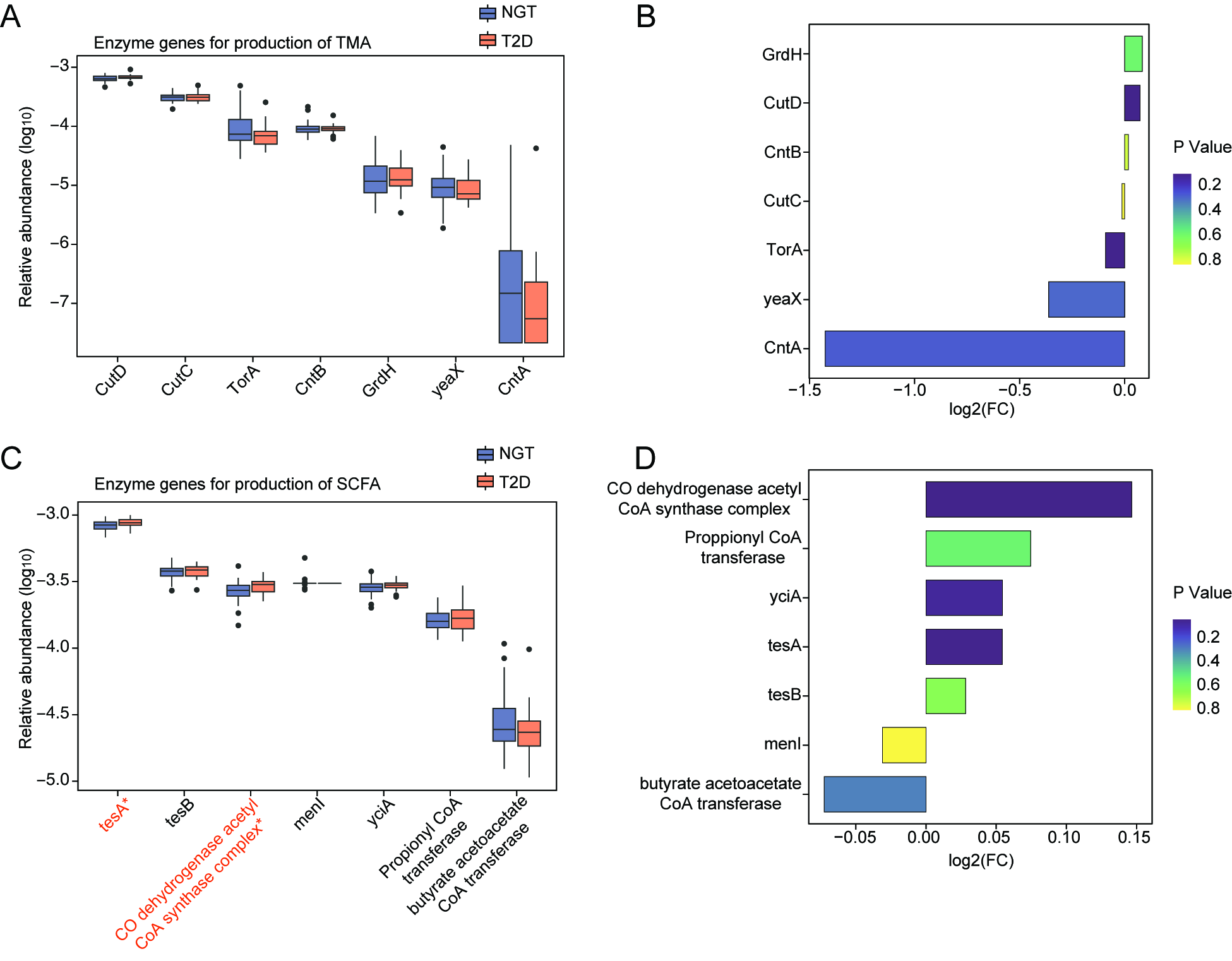

Supplement: Supplementary file 3 [file Image2.TIF]

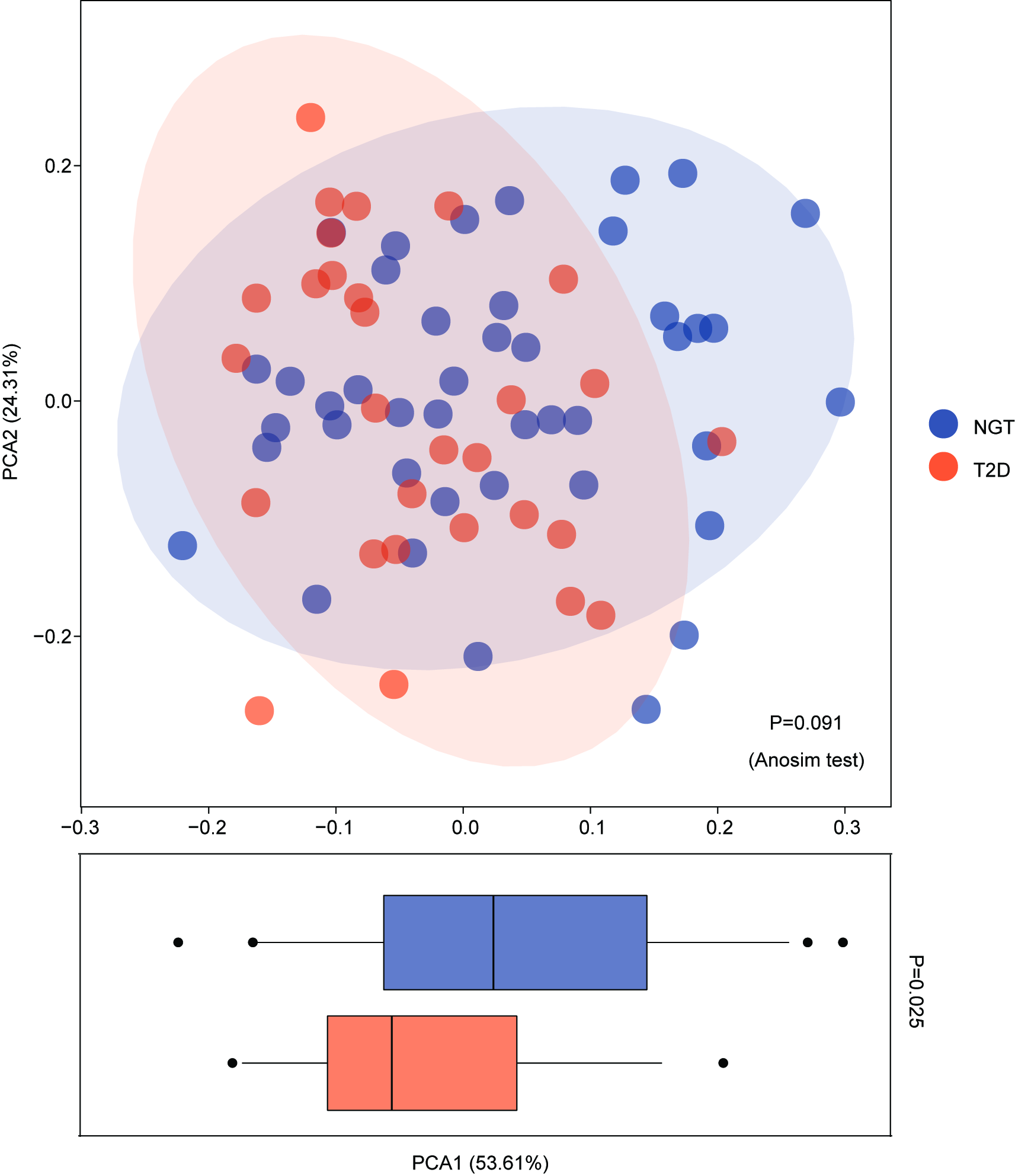

Supplement: Supplementary file 4 [file Image1.TIF]
