# Supplementary material for: Potential of gut microbiota for lipopolysaccharide biosynthesis in European women with type 2 diabetes based on metagenome
Source: Front Cell Dev Biol. 2022 Oct 11;10:1027413. doi: 10.3389/fcell.2022.1027413 (PMC9592851; doi:10.3389/fcell.2022.1027413)
Supplement: Supplementary file 5 [file DataSheet1.docx]

Supplementary Material

# Supplementary Figures and Tables

## Supplementary Figures

**
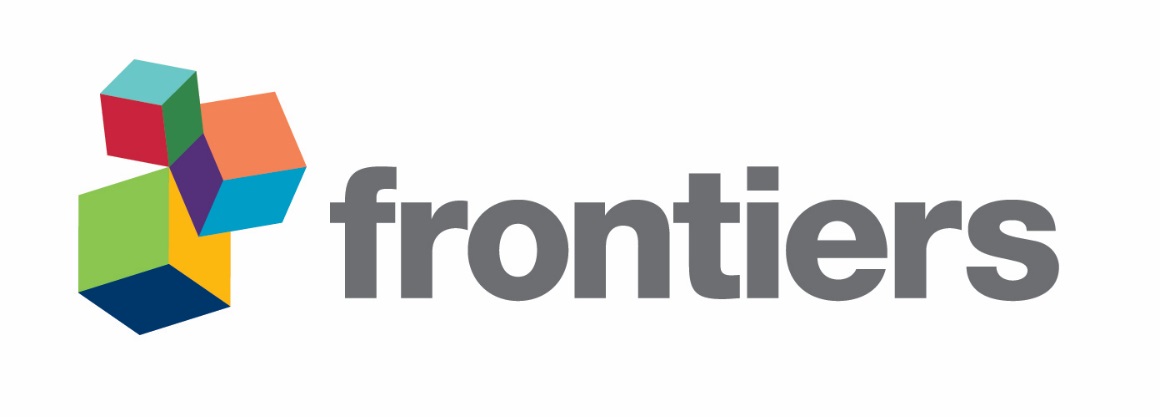
**

**Supplementary Figure 1.** PCA plot based on the abundance of nine LPS-biosynthesis-related enzymatic genes between NGT and T2D groups. P = 0.091 between groups was obtained using the ANOSIM test in PCA scatter plots. Significant differences were established at PCA1 between individuals with NGT and patients with T2D using the Wilcoxon rank-sum test. Circles in blue indicate samples from individuals with NGT, and circles in orange indicate samples from patients with T2D.

**Supplementary Figure 2.** Relative abundance of enzymatic genes for TMA-synthesis and SCFA-synthesis between individuals with NGT and patients with T2D. (A) Box plots showing the relative abundance of enzymatic genes for TMA-synthesis between patients with NGT and individuals with T2D (orange, T2D; blue, NGT). Boxes represent the interquartile ranges, the inside lines represent the median, and circles represent outliers. (B) Enrichment of TMA-biosynthesis-related enzymatic genes in groups was evaluated using Log2 (fold change T2D/NGT, FC). Log2 (FC) >0 and Log2 (FC) <0 indicate the enrichment in patients with T2D and individuals with NGT, respectively. The color varies from dark purple to light yellow, denoting the P value shift scale. (C) Relative abundance of enzymatic genes implicated in SCFA-synthesis between individuals with NGT and patients with T2D (orange, T2D; blue, NGT). (D) Enrichment of SCFA-biosynthesis-related enzymatic genes between groups as assessed using Log2 FC.

**Supplementary Figure 3.** Number of gut microbial species harboring LPS-biosynthesis-related enzymatic genes. Bar plots depicting the number of intestinal microbial species in the present cohort found to harbor genes for each LPS-biosynthesis-related enzyme at the, genus (purple), and species (green) levels.

## Supplementary Tables

**Supplementary Table 1.** Baseline characteristics of study population.
